# Identifying desired qualifications, tasks, and organizational characteristics of practice managers—a cross-sectional survey among group practice physicians in Germany

**DOI:** 10.1186/s12913-022-08199-5

**Published:** 2022-06-24

**Authors:** Clemens Schricker, Christoph Strumann, Jost Steinhäuser

**Affiliations:** University of Lübeck, Institute of Family Medicine, Ratzeburger Allee 160, 23538 Lübeck, Germany

**Keywords:** Practice management, Coordination of care, Primary care practice, Team practice, The delegation of tasks, Outpatient care

## Abstract

**Background:**

The increase of centralization developments in primary and secondary care practices may cause the organizational needs to increase as well, as the practices grow in size. This continuous change is observed in different stages in various countries since, from the perspective of a physician, it is reinforced by the benefits it adds to flexible work configuration, professional exchange and specialization. However, in order to benefit from the joint practice system, the proper managerial skills of practice managers are required, as doctors are not naturally prepared to fulfill such tasks. This study thus aims to gain insight into physicians' views in group practices and acquire a greater understanding of expectations towards practice management and the emerging role of practice managers (PM).

**Methods:**

A cross-sectional study design was employed which utilized an anonymous online questionnaire. In total, 3,456 physicians were invited to participate in the study between February 8^th^ and March 17^th^ 2021 by the Association of Statutory Health Insurance Physicians of Baden-Württemberg, Germany. Bivariate and multivariate analyses were applied to characterize the expectations of physicians towards practice management.

**Results:**

The survey yielded 329 replies (9,5%). 50% of the participating practices already had a PM employed. In general, these practices were larger than practices without a PM. Most physicians (85%) considered a medical background to be essential for the task of a PM. While practices without a PM considered it important for PMs to have medical qualifications, practices with a PM favored qualifications in business administration. 77.2% of physicians preferred to educate and recruit PMs out of their current practice staff. Competence in organizational tasks, such as coordination of tasks and quality management, was considered to be an essential skill of a PM and had the highest agreement levels among those surveyed, followed by staff management of non-physicians, billing, bookkeeping, staff management of physicians and recruiting. Based on multivariate regression analysis, larger practices valued the role of a PM more and were more likely to employ a PM. Notably, the effect that size had on these items was more substantial for generalists than specialists.

**Conclusions:**

The benefits and importance of PMs as well as the potential for delegation are recognized, in particular, by larger practices. The positive feelings that physicians who already employ PMs have towards their contribution to ambulatory care are even more significant. Pre-existing medical support staff has been identified to be the most desirable candidates for taking on the role of PM.

**Supplementary Information:**

The online version contains supplementary material available at 10.1186/s12913-022-08199-5.

## Background

A gradual shift in the structure of medical practice can be observed in several countries, such as the United States of America, Taiwan, Great Britain, Denmark, Italy and Germany. Larger group practices are increasingly replacing solo practices [1]. This development includes the consolidation of physicians into multispecialty groups [2].

Newly qualified German physicians prefer to work part-time and seek employment over being self-employed. This originates from a desire for more flexibility, fewer organizational tasks, and a focus on work-life balance [3–6]. Joint practices are thus more tailored to their demands, which is likewise the case in other nations [4, 5, 7, 8].

On the one hand, joint practices offer several synergies [9, 10]: Minimizing individual risk perception, flexible working hours, a more significant focus on medical practice, room for specialization, and (inter-) professional exchange. Furthermore, there are operative advantages due to a higher patient flow that increases efficiency and effectiveness in practice [11]. On the other hand, all the potential benefits of joint practice need to be secured by adapting practice management routines for the new requirements of the large-scale practices. Moreover, such new tasks require specialized knowledge and a dedicated role [12].

There is no standard definition of a practice manager (PM) in the German healthcare system. The title of PM is not officially regulated and neither are its qualifications. Previous studies comparing the educational possibilities of German PMs identified a proliferated landscape of educational options [13]. Generally, PM's core qualifications are in operational management, quality management, and human resource management [12]. These skills become relevant as the numbers of medical and other staff naturally grow with the number of physicians in joint practices, which creates a necessity for management on a larger scale. Physicians could take on these new management tasks themselves but are not naturally prepared for them, as their training typically focuses on being knowledgeable in medicine.

Competency based frameworks such as the CanMEDS Framework are used worldwide in under- and postgraduate training and included management and organizational topics [14]. In Germany, such elements were introduced ten years ago. There also has been growing interest in additional qualifications such as the Masters of Business Administration (MBA) to cover the blind spots in the medical educational framework. However, physicians are still relatively unprepared for managerial tasks. [15, 16]. Therefore, it can be hypothesized that they are unwilling to shift their focus from medical practice to management.

As the core of a physician’s field of expertise will likely remain to be medicine, it can be argued that the role of dedicated PMs will gain more importance. Furthermore, the specialized managerial knowledge of PMs will be required to be adequately prepared for the challenges of larger practices to reap the benefits these practices promise [17–19]. Other professionals, such as medical assistants, will therefore have to address the emerging managerial tasks, since they were already the primary target audience for PM-education programs in Germany. Regardless, specialized training can provide an adequate response to the emerging challenges of the new practice configuration currently developing in Germany and beyond [20]. Therefore, the expectations towards PMs in this changing environment and the demand of current group practices from a physicians' perspective are relevant for future developments.

This research paper aims to explore practice management from the perspective of physicians and identify the desired qualifications, relevant tasks and organizational characteristics for PMs in Germany. Furthermore, by researching the perspectives of physicians, a more complete picture of the evolving role of a PM can be gained in order to develop a more standardized profile for PMs.

## Methods

### Questionnaire

A cross-sectional study design was employed, which utilized an anonymous online questionnaire with 32 items to ascertain physicians' perspectives as potential employers of PMs. The questionnaire contained closed questions and questions with the option for open-ended answers. The questions focused on the qualifications, backgrounds, and skills of prospective PMs as well as tasks delegated to the role. Additionally, questions were included to find out what the minimum number of physicians and what the rate of patient turnover would have to be in order to justify a PM. Essential demographic characteristics were also collected (for further information see Additional File 1).

The questions built upon the explorative qualitative interviews with physicians, which contained information on potentially relevant practice management topics as well as a PM’s role [21]. The questionnaire was further informed by a comparison of PM’s possibilities for qualifications in three different countries [12]. The research team drew upon their experience when they initially created the questionnaire. As part of the research subject group itself, the experiences of a GP and a former consultant of the Association of Statutory Health Insurance Physicians in Baden-Württemberg were also drawn on when conceptualizing the questionnaire.

After creating the questionnaire, it was piloted within the Association of Statutory Health Insurance Physicians and the Institute of Family Medicine in Lübeck via a think-aloud process. This process included two general practitioners and four senior staff members in the association (one team lead, one department head and two division heads). The latter group has gathered a deep understanding of practice managerial aspects through a close exchange with practices regarding organizational procedures, legal aspects, personnel issues, and billing. The questionnaire was amended and selectively altered in tone and focus according to the received feedback.

The questionnaire’s target group were physicians in a southwestern federal state of Germany (Baden-Württemberg) located in practices consisting of more than three physicians. Such practices can be characterized as larger organizations within the practice landscape of this area. No specialists were excluded from the survey, since practice management is relevant for all kinds of specialists within the physician population, in addition to the fact that larger-scale practices may have multiple specialists present. Furthermore, all legal forms of cooperation present in German outpatient care were included.

The contact information for the questionnaire was sourced from the Association of Statutory Health Insurance Physicians of Baden-Württemberg, of which all physicians in the outpatient care sector must be members. The dataset thus included all physicians in the federal state who met the criteria of working at a practice that consisted of more than three physicians. 4.465 physicians out of about 21.000 that met the criteria. As the questionnaire was distributed via e-mail, the 3.456 physicians who had an e-mail address were selected for the online questionnaire. The initial contact was made on February 8^th^ 2021 via e-mail by the Association of Statutory Health Insurance Physicians of Baden-Württemberg, followed by two separate reminders in two-week intervals.

### Statistical analysis

Descriptive statistics were applied to explore the general prevalence of specific qualifications among PMs and indicate tasks delegated to the PM’s role. Frequencies were used for items which scaled nominally, while means and standard deviations were used for numerical discrete variables.

Additionally, a comparative analysis of groups within the total sample was conducted in order to explore the differences between physicians who worked in practices which employed a PM and those who worked in practices who did not. We considered practice size, specialty, the physician’s expectations and the general perceptions of the PM role, the desired qualification profile and the relevant tasks of PMs to be significant characteristics. The physicians were split into two groups of generalists and specialists. The generalists’ group included general practitioners and pediatrics [22, 23]. Physicians of other specialties were considered to be specialists. Practice size was used to divide the physicians into a group of practices smaller than ten physicians and practices of ten or more physicians. Differences between the groups were analyzed by employing the Pearson ${\chi }^{2}$-test or the Mann–Whitney U-test, depending on the scale of the considered variable. All tests of significance were corrected using the Bonferroni method to address the problem of multiple testing [24].

Finally, two multivariate regression analyses were applied to estimate the associations between physicians’ characteristics and perspectives, and (1) the assessment of the importance of a PM for the practice as well as (2) the likelihood, whether a PM was present or not. Since a six-point Likert scale measured the importance, an Ordinal Logistic Regression Model was estimated. In order to estimate the likelihood for the presence of a PM, we applied a Binary Logistic Regression Model. In both models, we specified the number of physicians in a practice, the specialty group (generalists vs specialists), and other covariables (i.e. concerns and chances regarding PM employment, tasks of PMs, and sociodemographic characteristics) as regression variables. Furthermore, for each regression, an interaction effect between the number of physicians in the practice and the specialty group was specified to analyze whether the latter moderated the effect of the practice size.

## Results

The survey yielded 329 replies after its closure on 17^th^ March 2021, equating to a response rate of 9.5%. The gender distribution within the survey was almost equal between males and females, with some respondents identifying as non-binary (Table 1). The respondents’ average age was 51.7 years. The years of experience in outpatient care was concurrent with the participants’ age at 13.4 years. The data showed a 50% split between practices that already had a PM and those that did not. The largest specialty were GP, followed by internal medicine. Grouping the specialties revealed that 37.1% of the respondents belonged to the generalist group.

**Table 1:** Demographic and structural information

Variable	all *n* = 329	
	n/obs	%
*Sociodemographic characteristics*		
Male	141/290	48.6
Female	138/290	47.6
Non-binary	11/290	3.8
Age (mean (s.d.^a^) in years)	51.7 (9.0)/292	
Years of experience (mean (s.d.^a^) in years)	13.4 (9.6)/292	
*Practice manager*		
present	163/327	49.8
not present	164/327	50.2
*Specialty*		
GP	91/275	33.1
Internal medicine	49/275	17.8
Gynecology	26/275	9.5
Orthopedics	23/275	8.4
Pediatrics	11/275	4.0
Radiologist	17/275	6.2
Other	58/275	21.1
*Specialty-Group*		
Generalists	102/275	37.1
Specialists	169/275	61.5

*Notes*: ^a^ Standard deviation

### Comparative Group Analysis

In Table 2, the mean values for practice size, the physicians’ expectations and general perceptions were shown for all participants and for groups of physicians working in practices with and without a PM. Practices where a PM was present were larger at a mean of 6.5 doctors, 14.4 medical staff, and 4.7 other staff members in contrast to practices without a PM which consisted of a mean of 4.3 doctors, 9.7 medical staff, 2.5 other staff members. The amount of staff members that justified a PM according to the participants were, however, significantly smaller in the group of physicians with an employed PM. Physicians with a PM had a lower average for their required number that justified a PM at a mean of 2.9 doctors and 6 additional medical staff, whereas the numbers for practices without a PM were at a higher mean of 3.6 doctors and 7.1 additional medical staff.

**Table 2:** Comparative group analysis – size, size requirements and general perceptions

Variable	All (*n* = 327)	PM not present (*n* = 164)	PM present (*n* = 163)	*p*-value*
*Practice Size*				
Number of physicians (mean)	5.4	4.3	6.5	< 0.001^a^
Number of medical assistants (mean)	12.1	9.7	14.4	< 0.001^a^
Number of non-medical personal (mean)	3.6	2.5	4.7	0.004^a^
*Specialty*				
Generalists (%)	37.1	40.4	34.3	n.s.^b^
Specialists (%)	61.5	58.1	64.2	n.s.^b^
*Practice Size that justifies a PM*				
Number of patients per quarter (mean)	2403	2325	2483	n.s.^a^
Number of physicians (mean)	3.2	3.6	2.9	< 0.001^a^
Number of non-physicians (mean)	6.6	7.1	6.0	0.003^a^
*Agreement with the following statements (6 strongly agree—1 strongly disagree)* (means)				
A functioning team does not need a practice manager	2.2	2.7	1.7	< 0.001^a^
A PM is important for the practice	5.2	4.6	5.8	< 0.001^a^
A PM can relieve my workload	5.4	5.1	5.8	< 0.001^a^
It is easy to release an employee for training as a PM	3.5	3.1	3.8	0.004^a^
A PM should continuously educate himself or herself	5.5	5.4	5.6	0.030^a^
I have concerns about delegating non-medical tasks	2.2	2.5	1.9	< 0.001^a^
*Agreement with the following statements (Yes in %)*				
A personal time gain is important for me to employ a PM	88.1	82.3	93.8	0.028^b^
A PM should also take on medical tasks	61.1	62.2	59.5	n.s.^b^
Management of materials is/would be the responsibility of the PM	88.0	93.8	82.1	0.026^b^
Do you prefer to recruit a practice employee as a PM?	77.2	80.1	74.1	n.s.^b^
Sharing a practice manager with other practices makes sense	23.7	28.6	19.3	n.s.^b^
Risk of conflict with external PMs (6: very high risk, … 1: very low risk) (mean)	3.7	4.0	3.4	0.020^a^

*Notes*: *Adjusted for multiple testing of the number of tested variables (i.e., 20) according to Bonferroni correction; ^a^Mann–Whitney U-test p-value; ^b^Pearson ${\chi }^{2}$-test p-value; n.s. not significant (after Bonferroni correction at the 5% level)

At the same time, practices agreed that regardless of a team’s pre-existing functioning capacity, a PM may still be required with an average of 2.2 on the six-point Likert scale. Therefore, the PMs’ general importance was considered to be high, with an average of 5.2. Participants disagreed about the ease of making time available for the staff to be trained in the first place (mean 3.5), while practices with an employed PM had fewer concerns. Both groups of practices rated the need for continuous education as necessary, but the practices which had a PM rated it slightly higher. Finally, relatively few concerns about non-medical task delegation were reported, with more favorable views towards PMs held by those working at practices where they were already present.

When considering the expected effects of PMs on physicians and their work, 88.1% reported that saving time is essential for a PM’s employment. Physicians with a PM rated the time-saving potential higher than other physicians (93.3% vs. 82.3%). When considering suitable practice managers, 77.2% preferred to educate and recruit PMs out of their current practice staff. The risk of conflict with external PMs was perceived as a slight risk. The practices without a PM present perceived risk more strongly.

Table 3 shows the results of a comparative group analysis of the necessary qualifications and educational backgrounds preferred for the role as well as the tasks of PMs. Most physicians (85%) rated a medical background as essential for the task of a PM. This was confirmed by the participants who, when given a choice to rate several potential qualification backgrounds as relevant, considered medical assistants (86.0%) and nurses (52.9%) with specialized training as the groups who were the most suitable for the role of PM. On the other hand, the preference for medical assistants without added training was much lower and ranked below the preferences for PMs recruited from business and public administration professions.

**Table 3:** Comparative group analysis – necessary qualifications and tasks for PMs

Variable	All (*n* = 327)	PM not present (*n* = 164)	PM present (*n* = 163)	*p*-value*
The PM should have previous medical education. (Yes in %)	85.0	88.4	81.4	n.s.^b^
*Necessary qualifications of PMs (Yes in %)*				
Medical assistant with special training	86.0	86.6	85.3	n.s.^b^
Nurse with special training	52.9	59.1	46.0	n.s.^b^
Experienced business administration specialist	37.7	31.1	44.8	n.s.^b^
Experienced public administration specialist	27.1	23.2	31.3	n.s.^b^
Business administration / public administration specialist	19.5	16.5	22.7	n.s.^b^
Medical assistant	19.5	24.4	14.7	n.s.^b^
Nurse	10.9	13.4	8.6	n.s.^b^
Other	10.6	11.0	10.4	n.s.^b^
*Tasks for PMs (6 strongly agree—1 strongly disagree)*				
Task coordination	5.5	5.5	5.6	n.s.^a^
Quality management	5.5	5.5	5.5	n.s.^a^
Staff management (non-physicians)	5.2	5.1	5.4	n.s.^a^
Billing	5.0	5.0	5.1	n.s.^a^
Bookkeeping	4.2	4.2	4.2	n.s.^a^
Recruiting of non-physicians	3.9	3.4	4.4	< 0.001^a^
Staff management (physicians)	3.3	3.2	3.4	n.s.^a^

*Notes*: *Adjusted for multiple testing of the number of tested variables (i.e., 16) according to Bonferroni correction; ^a^Mann–Whitney U-test p-value; ^b^Pearson ${\chi }^{2}$-test p-value; n.s. not significant (after Bonferroni correction at the 5% level)

Task coordination and quality management are the organizational tasks which have the highest agreement levels among those questioned. This is followed by staff management (i.e., the management of subordinates) of physicians and non-physicians, billing, bookkeeping and recruiting. Recruiting of non-physicians and staff management of physicians have the lowest agreement levels. Staff management of non-physicians and recruiting of non-physicians are potential tasks for a PM which have stronger agreements in practices with a PM present.

### Multivariate Associations with PM’s importance and likelihood of their presence

In Table 4, the results of two multivariate regression analyses are shown. In addition to the assessment of the importance of a PM for the practice, we also considered the likelihood of the presence of a PM as a dependent variable.

**Table 4:** Regression analysis

Variable	A PM is			
	important *(6 strongly agree—1 strongly disagree)*		present *(Yes/No)*	
	Ordinal Logit		Binary Logit	
*Sociodemographic and practice characteristics*				
Female	0.45	0.48*	0.55*	0.60*
Age	0.00	-0.00	-0.01	-0.01
Number of physicians in practice	0.11**	0.10***	0.18***	0.18***
Generalists	-0.54*	-0.32	-0.31	-0.10
Interaction effect: number of physicians in practice & Generalists		0.29***		0.38***
*Concerns and chances for PM employment*				
A PM can reduce my workload	1.76***	1.83***	0.98***	1.01***
I have concerns about delegating non-medical tasks	-0.22**	-0.26***	-0.21*	-0.24**
*Tasks for PMs*				
Staff management (non-physician)	0.37**	0.34**	-0.07	-0.11
Staff management physician	-0.02	-0.01	-0.01	0.01
Recruiting non-physicians	0.21**	0.21**	0.41***	0.41***
Bookkeeping	0.03	0.06	-0.22**	-0.22**
Billing	-0.14	-0.15	0.15	0.15
Task coordination	-0.12	-0.04	-0.20	-0.11
Quality management	0.21	0.18	-0.30	-0.36
Observations	256	256	256	256
LOGLIKE	-222.5	-217.8	-137.9	-132.7
AIC	481.1	473.5	303.9	295.5

*Notes*: Significance levels: *10%, **5%, ***1%

The estimated coefficients were very similar across the different models. The gender and the age of the physicians was not associated with either dependent variable. Physicians who believed that a PM had the potential to reduce their workload, evaluated the importance for the practice higher and were also more likely to work in a practice with a PM. Another determining factor for the acceptance of a PM was found to be the task of recruiting non-physicians. Differences between both models could be observed for the following tasks: staff management of non-physicians and bookkeeping. While staff management of non-physicians was positively associated with the importance of a PM, it had no correlation with the likelihood of the presence of a PM. Physicians who regarded bookkeeping as an important task for a PM were less likely to work in a practice with a PM.

The results of the estimated practice size effects underlined the previous finding that the bigger the practice size tended to be, the higher that practice rated the importance of a PM. The likelihood for the presence of a PM was also higher in larger practices. Further, both practice size effects were stronger for generalists.

## Discussion

This research aims to explore the expectations and perceptions of current group practices concerning practice management from a physicians’ perspective. The analysis identifies PMs’ desired qualifications, relevant tasks, and organizational characteristics. Thereby a more comprehensive picture of the evolving role of a PM can be obtained.

Our results show that larger practices rate practice management as more important than smaller ones. Larger practices are also more likely to employ a PM and, thus, share a more robust perception of the importance of a PM. This can be interpreted as a sign of a PM’s positive effect on the personal time and efficiency gains for physicians. Nevertheless, it also emphasizes the need for interpersonal relationship management and managerial tasks in larger practices [10, 13, 25, 26].

Furthermore, our regression results suggest that the likelihood for a PM and its rated importance increases for generalists more strongly with practice size than for specialists. A possible reason could be that the increase in practice sizes might introduce more complexity into the practices of generalists than in the practices of specialists. PMs thus seem more important for generalists if developing into more centralized practices. It is of note that practices, which employed a PM, had a smaller threshold with regards to the number of physicians and non-physician staff that justified the hiring of a PM than practices who did not. Physicians without experience regarding PMs might be overestimating the practice sizes whose management could benefit from a PM. This seems in line with the fact that physicians are primarily educated in medical tasks, not managerial endeavors [17]. There is also a preference for medical staff (nurses, medical assistants) to fill this role since a medical background is regarded as a necessary qualification for the position. This is in alignment with the current system, as pre-existing education programs are mostly tailored towards medical staff [12]. The fact that physicians consider medical staff to be the most suitable category to be a PM is intuitive, since physicians are the most familiar with them. While recruiting and continuously training a PM out of the pre-existing medical staff is seen as desirable, practices struggle with dedicating the time and resources necessary to do so. It can be argued that medical personnel is in similarly short supply as physicians are [27]. Therefore, operational needs infringe on the potential development of pre-existing staff into a PM. Simultaneously, allocating time for continuous education allows for tasks to be fulfilled more competently, empowering the medical staff acting as PM to question given structures independently and potentially enhancing their work [28].

However, larger practices and practices with a PM also prefer business administration and public administration specialists in addition to the medical staff for the role of PM. Having first-hand experience with PMs and their complex tasks may lead to a more positive view of managerial professionals taking on the role. This is in line with findings from the UK, where PMs do not predominantly require a medical background [12]. The potential professionalization of organizational structures within larger practices separates roles that are created for medical and managerial tasks. With its extensive networks and centralized structures, primary care in the UK shows what such specialized roles can look like. This is also true regarding the government paying PMs in Great Britain, while German physicians have to finance PMs themselves [29]. Regardless, our results show that medical staff is still the first choice for the role of PM among the participating physicians.

An international scoping review found that while group practices improve physicians’ quality of life, the higher stress caused by the more complex interpersonal/staff relationships is a disadvantage [25]. In a German context, it was confirmed that burnout in joint practices was higher than in solo practices [30]. While practice management was not a relevant factor, it can be argued that a competent PM might address this interpersonal element of joint practices. The curriculum of most PM education programs in Germany and other countries includes human resource management, potentially equipping PMs with the tools to relieve such stressors [12].

Our results also confirm that larger practices have fewer concerns about delegating non-medical tasks. Therefore, the delegation of operational tasks is central to the PM’s role. The presence of a PM positively affects the willingness to delegate tasks such as the recruitment and staff management of non-physicians. However, the recruitment and management of physicians as well as the bookkeeping are less likely to be delegated. Physicians still want to be personally involved in these critically important tasks. Generally speaking, task delegation requires the delegator and the recipient of the tasks to trust, collaborate and communicate with confidence in each other in order to benefit from the process [31–33].

Overall, the findings highlight that physicians have clear preferences regarding PMs' practical tasks and educational background, thus shaping the emergent role and who fulfills it.

Lastly, there are no differences between men and women in the data. This is surprising, as systematic differences in various dimensions between the sexes have been documented previously [34].

### Strengths and Limitations

There is little quantitative research done into practice management, the role of PM, and opinions towards it. Nevertheless, our results give insights into physicians’ perceptions when considering a PM for their practices. Overall, the study has yielded a rather low response rate of 9,5%, amounting to 329 replies. The response rate is within an expected range, considering the target audience [35]. The physician population was, however, resembled reasonably accurately regarding gender, age, and specialization [5, 36]. The response rate may have also been negatively affected by the coronavirus pandemic’s prevalence at the survey time. In addition, there was a high frequency of mail communication via the Association of Statutory Health Insurance Physicians’ official channels, imposing additional operational stress on physician practices in general and obscuring the survey request.

Male and female respondents provided equal representation within the survey, giving confidence to the findings in this regard. The risk of a self-selection bias exists, with maybe only those who are already interested in the subject participating in the survey. Furthermore, practices without e-mail were not able to attend the survey. Moreover, employers and employees were both included as participants. Therefore, it is not possible to separate the specific employer perspective in this regard. However, it can be assumed that regardless of their respective status, both groups of physicians would have experiences and opinions on the topic as a result of working in larger practices.

Lastly, there is the matter of considering internists to belong to the group of specialists. In 2020, 1929 (53%) of the 3609 internists have been practicing as primary care physicians in the federal state Baden-Württemberg [37]. However, we did not assign this specialty to the group of generalists, since their postgraduate training was based on hospital organ-centered specialist care [38]. To increase the robustness against this specification, we alternatively classified internists to the generalist group and obtained qualitatively very similar results.

### Next Steps

It might be rewarding to contrast the expectations physicians have of PMs with the curriculum of the institutions which are offering practice management qualifications. Another critical factor is understanding the effects of a PM on cost-effectivity and the monetary benefits of delegation in group practices. Finally, it would be of interest to quantify the possible model of cost reduction within the healthcare system due to the employment of PMs on a larger scale.

## Conclusions

Centralization developments offer physicians a more flexible work configuration, professional exchange, and specialization in ambulatory care practices. At the same time, the increased organizational needs of larger practices necessitate the establishment of practice management routines. The physicians surveyed in this study recognized the benefits of employing a PM. As a result, PMs’ acceptance is higher and the presence of PMs is more frequent in larger practices than in smaller ones. The task delegation is also more prevalent in practices with a PM. Physicians, however, are hesitant to delegate critical tasks such as the billing and management of the physician staff. Regardless, specialized education is required to enable medical or administrative staff to take on management tasks and alleviate operational and interpersonal pressure from physicians. The purpose of a PM is, after all, to realize managerial potential and alleviate tasks from physicians.

## Supplementary Information

**Additional file 1.** Translated_Questionnaire. The original German version of the questionnaire was used to identify desired qualifications, tasks, and organizational characteristics of practice managers as part of the cross-sectional survey among group practice physicians in Germany.

## Data Availability

The data that supports the findings of this study is available from the authors, but restrictions apply to the availability of this data, which was used under license for the current study, and so are not publicly available. However, the data is available from the authors upon reasonable request and with permission of the Association of Statutory Health Insurance Physicians in Baden-Württemberg.

## Abbreviations

FTE: Full-time equivalents
WLB: Work-life balance
PM: Practice manager
