# Supplementary material for: Identifying desired qualifications, tasks, and organizational characteristics of practice managers—a cross-sectional survey among group practice physicians in Germany
Source: BMC Health Serv Res. 2022 Jun 24;22:821. doi: 10.1186/s12913-022-08199-5 (PMC9233318; doi:10.1186/s12913-022-08199-5)
Supplement: Supplementary file 1 — Additional file 1. Translated_Questionnaire. The original German version of the questionnaire was used to identify desired qualifications, tasks, and organizational characteristics of practice managers as part of the cross-sectional survey among group practice physicians in Germany. [file 12913_2022_8199_MOESM1_ESM.pdf]

Appendix 1: A translated questionnaire used to identify desired qualifications, tasks, and organizational characteristics of practice managers as part of the cross-sectional survey among group practice physicians in Germany

No.	Question	Reply
1	A Personal time gain is important for me to employ a practice manager.	Yes/No
2	Does your practice have a practice manager?	Yes/No
3	The practice manager should have previous medical training.	Yes (Free Text for Specification)/No
4	A PM should also take on medical tasks	Yes/No
5	Management of materials (purchasing, procurement, inventory management, etc.) is/would be the responsibility of the PM.	Yes/No
6	What basic training do you see as necessary qualifications of a practice manager?	Multiple answers:<br><br>Medical assistant with special training/Nurse with special training/Experienced business administration specialist/Experienced public administration specialist/Business administration/public Administration specialist/Medical assistant/Nurse/Other
7	Do you prefer to recruit a practice employee as a PM or external person?	Employees of the practice / external person
8	If an employee of the practice has been selected as practice manager: It is easy to release an employee for training as a practice manager.	fully agree – strongly disagree<br><br>(6-point scale)
9a	I think a practice manager is important for the practice.	fully agree – strongly disagree<br><br>(6-point scale)
9b	A practice manager can relieve my workload.	fully agree – strongly disagree<br><br>(6-point scale)
10	A practice manager should continuously educate himself or herself.	fully agree – strongly disagree<br><br>(6-point scale)
11	The staff management of non-physicians staff (duty rosters, vacation planning, etc.) is the task of the practice manager.	fully agree – strongly disagree<br><br>(6-point scale)
12	The staff management of physician staff (duty rosters, vacation planning, etc.) is the task of the practice manager.	fully agree – strongly disagree<br><br>(6-point scale)
13	Selection and recruiting of non-physicians staff (interviews, screening of applications) is the responsibility of the practice manager	fully agree – strongly disagree<br><br>(6-point scale)
14	Bookkeeping (paying bills and salaries, managing receivables, etc.) is the responsibility of the practice manager.	fully agree – strongly disagree<br><br>(6-point scale)
15	Billing (e.g., track billing for services) is the responsibility of the practice manager.	fully agree – strongly disagree<br><br>(6-point scale)
16	Operative coordination of practice procedures (organization of standardized processes,	fully agree – strongly disagree<br><br>(6-point scale)

	implementation of regulations and control, etc.) is the task of the practice manager.	
17	The implementation of quality management (fulfillment of documentation obligations, evaluation of processes, etc.) is the task of the practice manager.	fully agree – strongly disagree<br>(6-point scale)
18	I have concerns about delegating non-medical tasks.	fully agree – strongly disagree<br>(6-point scale)
19	A functioning team does not need a practice manager.	fully agree – strongly disagree<br>(6-point scale)
20	How high do you assess the risk of an external practice manager not being accepted by the other employees?	Very high risk – very low risk<br>(6-point scale)
21	Sharing a practice manager with other practices makes sense.	Yes/No
22	How do you estimate the time required for coordination with the practice manager?	Time in minutes/day
23	What do you consider a justifiably gross salary for a practice manager per year?	Wage in thousand euros
24	From what number of patients per quarter with statutory health insurance do you consider a practice manager to be necessary?	Free text (in numbers)
25	From what number of physicians (employed and licensed) do you consider a practice manager to be necessary?	Number – Dropdown (1,2,3,4,5,6,7,8,9,>10)
26	From what number of non-physicians (e.g., nurses) do you consider a practice manager to be necessary?	Number – Dropdown (1,2,3,4,5,6,7,8,9,>10)
27	What gender are you?	Male, female, non-binary
28	How old are you?	Number – Dropdown
29	How many physicians work (including you) in your practice?	Number – Dropdown
30	How many medical assistants work in your practice?	Number – Dropdown
31	How many other employees (e.g., cleaners) work in your practice?	Number – Dropdown
32	How many years of experience do you have working3 in practices?	Number – Dropdown
33	Which specialist title do you hold?	Selection of medical specialties
34	Beyond the questions, do you have any points you would like to share regarding the topic?	Free text
